# Thermal Studies of Zn(II), Cd(II) and Hg(II) Complexes of Some *N*-Alkyl-*N*-Phenyl-Dithiocarbamates

**DOI:** 10.3390/ijms13089502

**Published:** 2012-07-27

**Authors:** Damian C. Onwudiwe, Peter A. Ajibade

**Affiliations:** Department of Chemistry, University of Fort Hare, Private Bag X1314, Alice 5700, South Africa; E-Mail: donwudiwe@ufh.ac.za

**Keywords:** Group 12 metals, dithiocarbamates, thermal behavior, differential scanning calorimetry, thermogravimetric analysis

## Abstract

The thermal decomposition of **Zn(II)**, **Cd(II)** and **Hg(II)** complexes of *N*-ethyl-*N*-phenyl and *N*-butyl-*N*-phenyl dithiocarbamates have been studied using thermogravimetric analysis (TGA) and differential scanning calorimetry (DSC). The products of the decomposition, at two different temperatures, were further characterized by scanning electron microscopy (SEM) and energy-dispersive X-ray spectroscopy (EDX). The results show that while the zinc and cadmium complexes undergo decomposition to form metal sulphides, and further undergo oxidation forming metal oxides as final products, the mercury complexes gave unstable volatiles as the final product.

## 1. Introduction

Dithiocarbamates are S, N containing ligands, which display a rich and varied coordination chemistry with a wide range of transition and main group metal complexes [1]. The chemistry of these compounds has aroused special interest because of the analytical purpose, as well as their industrial applicability [2,3]. Their metal complexes present striking structural features and have diversified applications, such as high pressure lubricants in industry, fungicides and pesticides, and also as accelerators in vulcanization [4]. Dithiocarbamate complexes constitute one of the most promising species to provide single-source materials for bulk metal sulfides [5,6]. Dithiocarbamates have also found important use in medicine as anti-alcoholic drug [7], anticancer [8], and recently as co-adjuvant in AIDS treatment [9]. This is related to their strong metal binding capacity, hence could act as inhibitors of enzymes [10]. Thermo analytical methods are of great interest due to their wide applicability in the industrial processes that involves the thermal decomposition reactions of solids [11,12]. Hence research in this area has gained increased attention [13].

The thermochemistry of metal dithiocarbamates has been widely studied in the past years but little is known about their thermal decomposition kinetics [14–16]. The TGA revealed that generally these complexes either volatilize leaving a negligible amount of residue or decompose to yield metal sulphide [17]. Most of the dithiocarbamates studied are prepared from aliphatic amines [18] and little is known about the thermal studies of the alkyl-aryl dithiocarbamates. The aim of this work is to study the thermal and kinetic behavior of *N*-alkyl-*N*-phenyl dithiocarbamate complexes of Zn(II), Cd(II) and Hg(II) (alkyl = ethyl and butyl) using the non-isothermal method.

## 2. Results and Discussion

### 2.1. Thermal Decomposition

**Figure 1:**
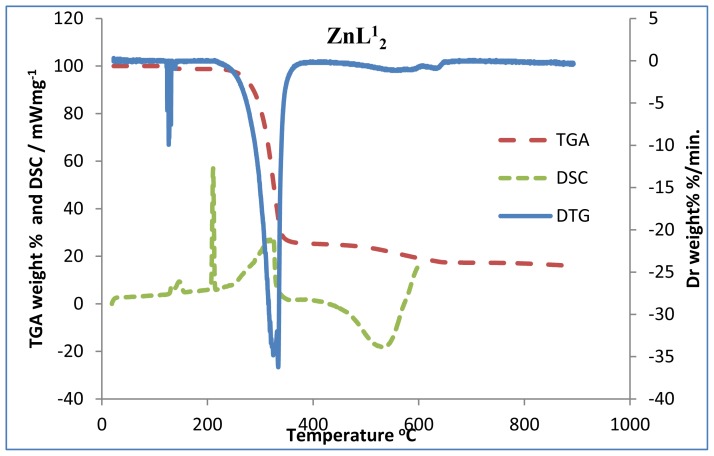

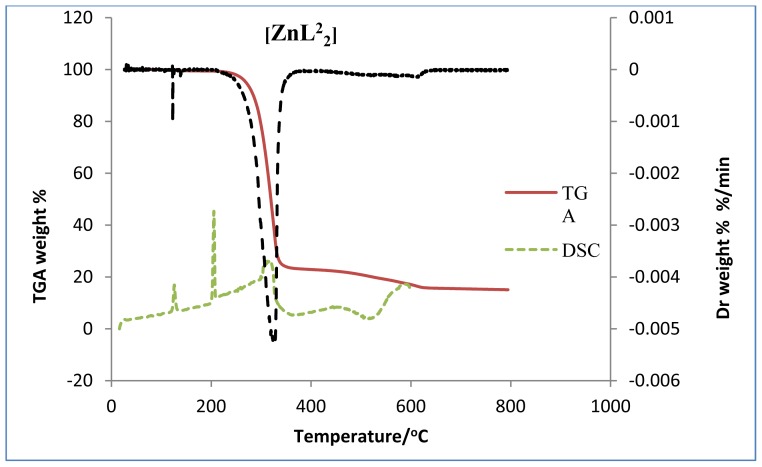
Superimposed thermogravimetric (TG), differential thermogravimetric (DTG) and differential scanning calorimetric (DSC) curves for zinc complexes (ZnL^1^_2_ and ZnL^2^_2_).

**Figure 2:**
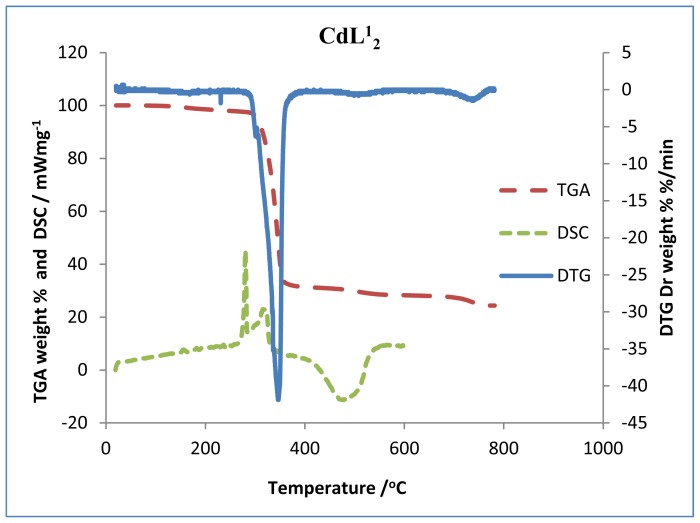

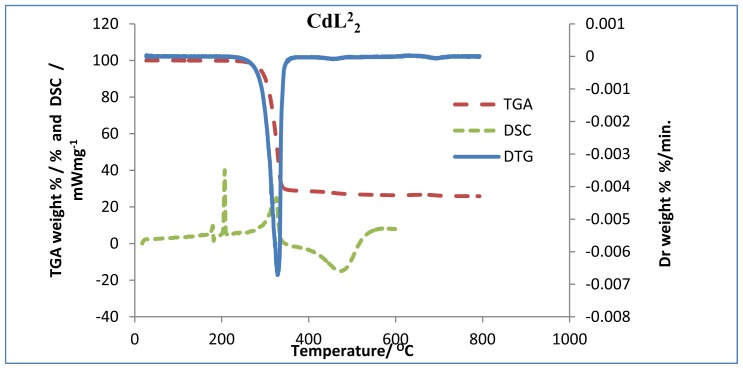
Superimposed TG/DTG/DSC curves for cadmium complexes (CdL^1^_2_ and CdL^2^_2_).

**Figure 3:**
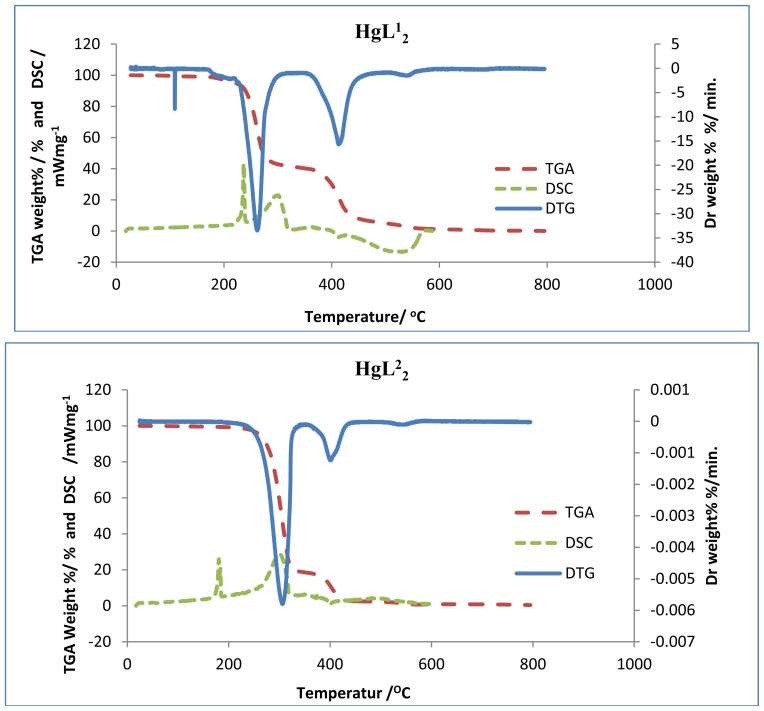
Superimposed TG/DTG/DSC curves for mercury complexes (HgL^1^_2_ and HgL^2^_2_).

Thermogravimetric (TG), differential thermogravimetric (DTG) and differential scanning calorimetric (DSC) analysis were carried out for the complexes under nitrogen flow. Figures 1–3 show the TG/DTG/DSC curves for all the complexes, while Table 1 contains the maximum temperature values for decomposition along with corresponding weight loss values.

The TGA curves indicate that the loss of weight starts around 220 °C for the zinc complexes, and at much higher temperature for the cadmium complexes. The two mercury complexes show a wide difference in their onset decomposition temperature. While bis(*N*-ethyl-*N*-phenyl dithiocarbamate) mercury (II) commenced decomposition around 180 °C, bis(*N*-butyl-*N*-phenyl dithiocarbamate) mercury (II) remained stable until about 211 °C. The decomposition continues to about 360 °C at which point most of the organic part of the compounds have been lost. This sharp decomposition period brings about 73%–76% weight loss in the zinc complexes, 66%–70% weight loss in the cadmium complexes and 57%–80% weight loss in the mercury complexes; and led to the complete formation of sulphide [19]. Zinc (II), cadmium (II), and mercury (II) diethyldithiocarbamates represent a class of volatile dithiocarbamates yielding the corresponding metal sulphide at about 623 °C [20,21]. We have noticed a shorter decomposition temperature for these complexes and this could be ascribed to the effect of the aryl group. The mercury complexes show three decomposition peaks in the thermogram. The first decomposition peak, which has the highest weight loss, corresponds to the formation of the HgS. In accordance with stoichiometric calculation, the product of the second decomposition step for the mercury complexes is possibly sulphur. The DTG indicate the high volatility of the first decomposition product (HgS), in this temperature region. The literature reports support the identification of the volatile decomposition product as Hg vapour and sulphur. Hardy, *et al.* [22] has reported that the product of HgS heated in the air is mercuric oxide, which decomposes to mercury vapour and oxygen at about 500 °C. Also, in a process sometimes referred to as reactive sublimation, the decomposition of HgS has been identified to proceed according to the chemical reaction:

(1) $${\text{HgS}}_{\left(\text{S}\right)}\to {\text{Hg}}_{\left(\text{g}\right)}+1/2\;{\text{S}}_{2(\text{g})}$$

The third peak around 550 °C could be ascribed to the oxidation of the sulphur to sulphur dioxide (SO_2_). By comparing the TG and DSC curves, it showed that the thermal decomposition began at temperatures higher than the melting point of the compounds, and so occurred in the liquid phase. This occurred in a single-step process as suggested by the DTG curves. Certain factors may be said to be responsible for the slight difference in the weight values of the end product (Table 1). The presence of oxygen and sulphur in the end product of the zinc and cadmium complexes at 800 °C, as shown by the EDX result, may indicate oxysulphate which probably have formed due to the oxidation of the sulphide.

The complexes showed similar behaviours in the DSC. There are two sharp endothermic peaks between 200 °C–320 °C and one broad exothermic peak between 400 °C–550 °C, except in the mercury complexes where small exothermic peaks are observed at 398 °C and 378 °C for HgL^1^_2_ and HgL^2^_2_ respectively. An additional broad peak is observed at 507 °C for HgL^1^_2_. The first endothermic peaks correspond to the melting of the complexes while the second represent their decomposition to form the respective metal sulphides. A striking feature of the DSC peaks is the progressive decrease in temperature with increase in number of the carbon atoms of the alkyl substituent for a particular metal ion. This trend is also observed down the group (Zn < Cd < Hg) and could be ascribed to the increase in molecular weight and also, the increase in the size of the central metal ion respectively. A reduction trend is also observed in the broad exothermic peaks of the zinc and cadmium complexes. The broad exothermic peaks may presumably be due to the oxidation of the metal sulphides to metal oxide (ZnO, CdO, and HgO) [23]. It is apparent that two different steps of decomposition are associated with the degradation of the complex and the residue, the later being an oxidation reaction. The DSC curves show that before the volatilization of the mercury complex, oxidation did actually occur. From the results of the TGA and DSC, one can recognize the order of decreasing stability of the complexes as follows: Cd < Zn < Hg.

**Scheme 1:**
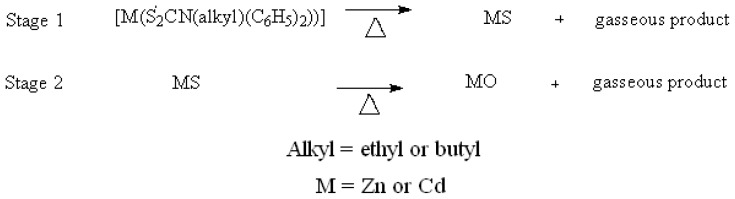
Decomposition pattern for the zinc and cadmium complexes.

The probable decomposition pattern for the zinc and cadmium complexes could be schematically represented as shown in scheme 1.

### 2.2. SEM/EDX Analyses

**Figure 4:**
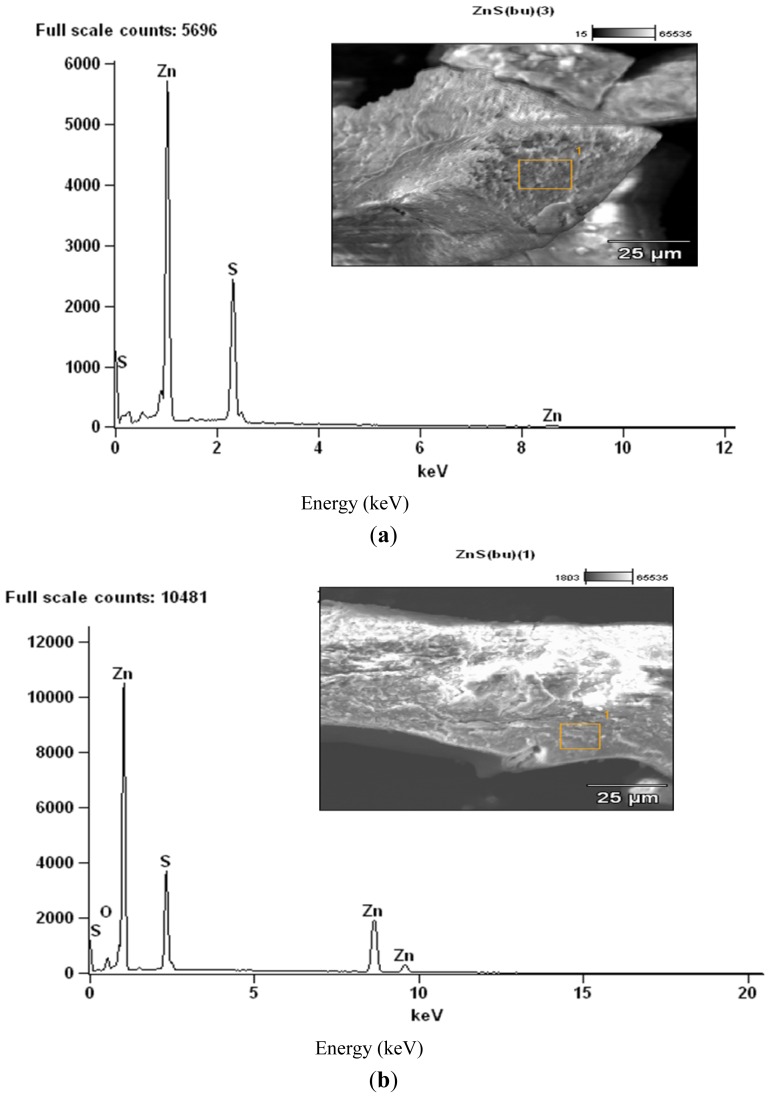
(**a**) Energy dispersive X-ray analysis (EDX) of the decomposed products from complex ZnL^2^_2_ at 400 °C; (**b**) EDX of the decomposed products from complex ZnL^2^_2_ at 800 °C.

**Figure 5:**
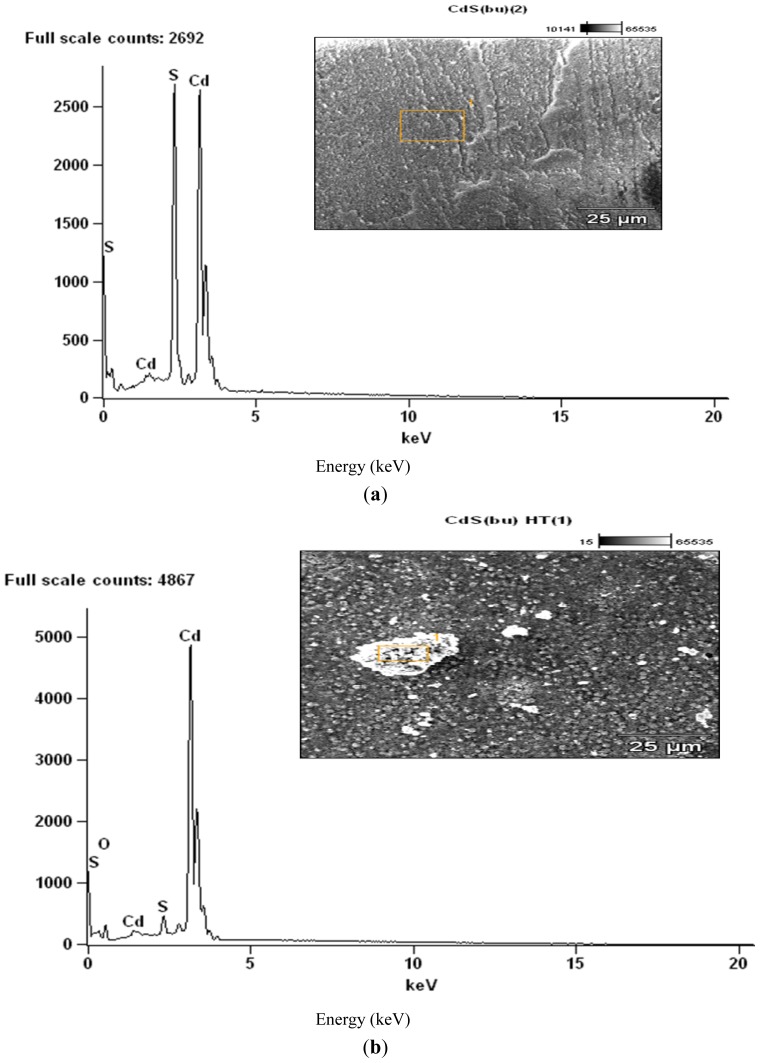
(**a**) EDX of the decomposed products from complex CdL^2^_2_ at 400 °C; (**b**) EDX of the decomposed products from complex CdL^2^_2_ at 800 °C.

**Figure 6:**
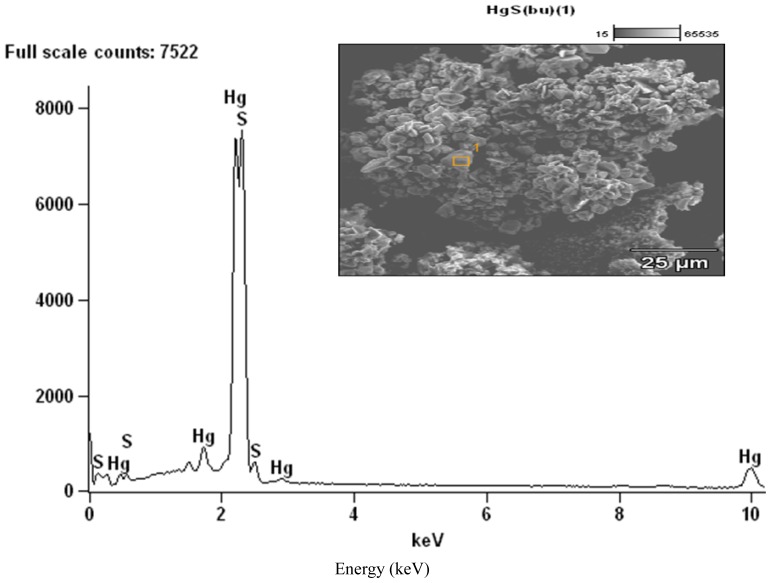
EDX of the decomposed products from complex HgL^2^_2_ at 350 °C.

The residues obtained from these decompositions were subjected to SEM/EDX analyses (Figures 4–6). The complexes exhibited different morphologies and colours with respect to temperature gradient. All complexes were powdered samples at the onset of the decomposition. At the end of the first stage of the decomposition, the zinc sulphide was obtained as shiny black crystalline blocks, which changed into fine black powdered samples at the end of the heating process to 800 °C. The cadmium complexes gave pale yellowish-green solid at the end of the first decomposition temperature but as the temperature increased the solid changed further to orange brown and finally to dirty yellow powder, probably due to partial oxidation of the sulphide. The SEM image of the cadmium complexes at 800 °C, taken at high magnification, shows some light patches indicative of the presence of sulphates probably due to partial oxidation of the sulphide.

The EDX analyses showed that at 400 °C, the products obtained for the Zn and Cd were the respective metal sulphides (Figures 4a and 5a) while at the end of the decomposition, at 800 °C (Figures 4b and 5b), oxygen peaks were observed and these suggest the presence of sulphate or sulphides which are likely product of oxidation of the metal sulphide. Due to the volatility of the HgS at the second stage of decomposition, the EDX of the residue could not be obtained at the end of the decomposition at 800 °C.

## 3. Experimental Section

### 3.1. Sample Preparation and Characterization

The synthesis and characterization of the ligands, *N*-ethyl-*N*-phenyl dithiocarbamate [(C_2_H_5_)(C_6_H_5_)NCS_2_-] and *N*-butyl-*N*-phenyl dithiocarbamate [(C_4_H_9_)(C_6_H_5_)NCS_2_-]; and their respective metal complexes [((C_2_H_5_)(C_6_H_5_)NCS_2_)_2_M] and [((C_4_H_9_)(C_6_H_5_)NCS_2_)_2_M] (M = Zn, Cd and Hg) studied in this work are described in detail elsewhere [24]. The ligands were prepared under ice temperature while the metal-complexes reported were prepared by the reaction of their respective metal chlorides with the ligands in 1:2 molar ratios in aqueous solution at room temperature.

### 3.2. Thermal Measurements

Perkin Elmer thermogravimetric analyzer (TGA 7) equipped with a thermal analysis controller (TAC 7/DX) was utilized. For each experiment, around 10–12.00 mg of the prepared complexes sample was used. The heating rate was 10 °C·min^−1^, the temperature ranging from 20 °C to 800 °C and the purge gas was nitrogen maintained at a flow rate of 20 mL·min^−1^. The differential-scanning calorimetry at high temperature was performed with a Thermo scientific DSC (i–series) instrument for temperatures ranging from 20 to 600 °C at a rate of 5 °C·min^−1^, in nitrogen atmosphere.

### 3.3. Scanning Electron Microscopy (SEM) and EDX Analysis

The SEM images were obtained in a Jeol, JSM-6390LV apparatus, using an accelerating voltage between 15–20 kV at different magnifications. Composition and energy dispersive spectra were processed using energy dispersive X-ray analysis (EDX) attached to the SEM with Noran System six software. For the EDX analysis, an accelerating voltage of 20.0 KV and magnification of 1000 were used.

## 4. Conclusions

The thermal decomposition and kinetics of Zn(II), Cd(II) and Hg(II) complexes of *N*-alkyl-*N*-phenyl dithiocarbamate has been investigated by the combination of TGA/DSC analysis and SEM/EDX. The thermal decomposition behaviour of the complexes proceeds in one major decomposition step to give the respective metal sulphides. A second step of exothermic decomposition occurred at higher temperature which has been ascribed to the oxidation of the sulphides. The thermal decomposition studies have shown the formation of metal oxide as the final product, except for in the Hg complexes where volatilization took place.

## Figures and Tables

**Table 1:** Temperature values for decomposition along with corresponding weight loss values.

Compounds	Decomposition range (°C)	Peak temperature (°C)	Percentageweight loss (%)	Productexpected	Residue state/colour	Mass changesCalc. (Found)
[Zn(S_2_CN(C_2_H_5_)(C_6_H_5_))_2_]ZnL^1^_2_	496–631	59	73.7	ZnS	Black crystals	2.50 (2.90)
		4809	81.8	ZnO	Grey ash powder	2.07 (2.04)

[Cd(S_2_CN(C_2_H_5_)(C_6_H_5_))_2_]CdL^1^_2_	542–634	606	66.7	CdS	Yellowish-green powder	3.07 (3.10)
		761	69.8	CdO	Brown powder	2.72 (2.14)

[Hg(S_2_CN(C_2_H_5_)(C_6_H_5_))_2_]HgL^1^_2_	453–591	532	57.3	HgS	Black powder	4.13 (4.01)
		683	97.8	S	-	0.56 (0.55)

[Zn(S_2_CN(C_4_H_9_)(C_6_H_5_))_2_]ZnL^2^_2_	494–644	591	76.1	ZnS	Black crystals	1.80 (2.10)
		861	83.6	ZnO	Grey ash powder	1.50 (1.50)

[Cd(S_2_CN(C_4_H_9_)(C_6_H_5_))_2_]CdL^2^_2_	503–642	599	70.8	CdS	Yellowish-green powder	3.88 (3.24)
		709	72.5	CdO	Yellowish brown powder	2.56 (2.90)

[Hg(S_2_CN(C_4_H_9_)(C_6_H_5_))_2_]HgL^2^_2_	484–618	575	80.3	HgS	Black powder	1.80 (1.85)
		669	97.3	S	-	0.27 (0.29)

## References

[1] Bajpai A., Tiwari S. (2004). Application of thermogravimetric analysis for characterization of bisdithiocarbamate of urea and its copper (II) complex. Thermochim. Acta.

[2] Scarcia V., Furlani A., Fregona D., Faraglia G., Sitran S. (1999). Palladium and platinum dithiocarbamato complexes containing mono-and diamines. Polyhedron.

[3] Singha A., Dutta D.P., Tyagi A.K., Mobin S.M., Mathur P., Lieberwirth I. (2007). Palladium(II)/allylpalladium(II) complexes with xanthate ligands: Single-source precursors for the generation of palladium sulfide nanocrystals. J. Organom. Chem.

[4] Ondrušová D., Jóna E., Šimon P. (2002). Thermal properties of *N*-ethyl-*N*-phenyl-dithiocarbamates and their influence on the kinetics of cure. J. Therm. Anal.

[5] Ali B.F., Al-Akramawi W.S., Al-Obaidi K.H., Al-Karboli A.H. (2004). A thermal analysis study of dialkyldithiocarbamato nickel(II) and copper(II) complexes. Thermochim. Acta.

[6] O’Brien P., Walshm J.R., Watson I.M., Motevalli M., Henriksen L (1996). Novel dithio- and diseleno-carbamates of zinc and cadmium as single-molecule precursors for low-pressure metal-organic chemical vapour deposition. J. Chem. Soc. Dalton Trans.

[7] Vettorazzi G., Almeida W.F., Burin G.J., Jaeger R.B., Puga F.R., Rahde A.F., Reyes F.G., Schvartsman S. (1995). International safety assessment of pesticides: Dithiocarbamate pesticides, ETU and PTU A review and update. Teratog. Carcinog. Mutag.

[8] Milacic V., Chen D., Ronconi L., Landis-Piwowar K.R., Fregona D., Dou Q.P. (2006). A novel anticancer gold(III) dithiocarbamate compound inhibits the activity of a purified 20S proteasome and 26S proteasome in human breast cancer cell cultures and xenografts. Cancer Res.

[9] Rabbi M.F., Finnegan A., Al-Hartli L., Stong S., Roebuck K.A. (1998). Interleukin-10 enhances tumor necrosis factor-α activation of HIV-1 transcription in latently infected T cells. Acquired Immune Defic Syndr. Hum. Retrovirol.

[10] Malik A.K., Faubel W. (1999). Methods of analysis of dithiocarbamate pesticides: A review. Pestic Sci.

[11] Santos J.G., Conceição M.M., Trindade M.F.S., Araújo A.S., Fernandes V.J., Souza A.G. (2004). Kinetic study of dipivaloylmethane by Ozawa method. J. Therm. Anal. Calorim..

[12] Conceição M.M., Ouriques H.R.C., Trindade M.F.S., Prasad S., Filho P.F.A., Souza A.G. (2004). Kinetics of decomposition of alkylammonium salts. J. Therm. Anal. Calorim.

[13] Souza A.G., Oliveira M.M., Santos I.M.G., Conceição M.M., Nunes L.M., Machado J.C. (2002). Kinetic analysis of the thermal decomposition of dialkyldithiocarbamates chelates of indium(III). J. Therm. Anal. Calorim.

[14] Dias S.C., Brasilino M.G.A., Pinheiro C.D., Souza A.G. (1994). Metal—Sulphur bond enthalpy determination of diethyldithiocarbamate complexes of cadmium and mercury. Thermochim. Acta.

[15] Carvalho M.A.P., Airoldi C., Souza A.G. (1992). Thermochemical features of di-*N*-propyldithiocabamate chelates of zinc-group elements. J. Chem. Soc. Dalton Trans.

[16] Souza A.G., Melo A.M.S., Santos L.C.R., Espínola J.G.P., Oliveira S.F., Airoldi C. (1998). Standard molar enthalpies of formation and lattice energies of 1-methylethyl, *N*-butyl and 2-methylpropyl ammonium halides. Thermochim. Acta.

[17] Siddiqi K.S., Nami S.A.A., Lutfullah, Chebude Y. (2006). Template synthesis of symmetrical transition metal dithiocarbamates. J. Braz. Chem. Soc..

[18] Szafranek A., Szafranek J. (1995). Thermogravimetric studies of alkyl *N*-aryldithiocarbamates. Thermochim. Acta.

[19] Khan S., Nami S.A.A., Siddiqi K.S. (2008). Piperazine pivoted transition metal dithiocarbamates. J. Mol. Struct.

[20] D’Ascenzo G., Wendlendt W.W. (1969). The thermal properties of some metal complexes of diethyldithiocarbamic acid new volatile metal chelates. J. Therm. Anal.

[21] D’Ascenzo G., Wendlendt W.W. (1970). Iron(III) diethyldithiocarbamate—A new volatile metal chelate. J. Inorg. Nucl. Chem.

[22] Hardy A.D., Sutherland H.H., Vaishanav R., Worthing M.A. (1995). A report on the composition of mercurials used in traditional medicines in Oman. J. Ethropharmacol.

[23] Singhal S., Garg A.N., Chandra K. (2007). Synthesis of tris(*N*,*N*′-dialkyldithiocarbamato) iron(III) complexes and their thermal decomposition studies by various techniques. J. Alloys Compd.

[24] Onwudiwe D.C., Ajibade P.A. (2010). Synthesis and characterization of metal complexes of *N*-alkyl-*N*-phenyl dithiocarbamates. Polyhedron.

